# Dramatic thrombolysis after rapid injection of tissue plasminogen activator: A case report

**DOI:** 10.1097/MD.0000000000017331

**Published:** 2019-09-20

**Authors:** Jin-Sung Park, Jaechun Hwang

**Affiliations:** Department of Neurology, School of Medicine, Kyungpook National University, Kyungpook National University Chilgok Hospital, Daegu, South Korea.

**Keywords:** intravenous rt-PA, ischemic stroke, rapid recanalization, thrombolysis

## Abstract

**Rationale:**

The regimen of the recombinant tissue plasminogen activator (rt-PA) is identical in every case where it is indicated in the treatment of cerebral infarction. We report a case of efficient recanalization of large arterial occlusion after rapid injection of rt-PA.

**Patient concerns:**

A 78-year-old man was admitted with right-sided hemiplegia and global aphasia that occurred an hour ago.

**Diagnoses:**

His brain computed tomography (CT) revealed no hemorrhage, suggesting cerebral infarction.

**Interventions:**

Ten percent of a total rt-PA dose was injected over 1 minute promptly. The remainder of rt-PA was designed to be infused for 60 minutes. Unexpectedly, during the study of CT angiography, administration of rt-PA was completed within 5 minutes. CT angiography showed occlusion from carotid bifurcation to the middle cerebral artery.

**Outcomes:**

After 2 hours of rt-PA administration, the patient began to regain strength in his right arm and leg. By the next day, he had only mild dysarthria and aphasia. Follow-up CT angiography revealed recanalized internal cervical artery and severe residual stenosis with a plaque. He was discharged without any neurologic symptoms.

**Lessons:**

The infusion protocol of rt-PA administration is established in 1995 and has not changed. Successful recanalization of long segmental large vessel occlusion with only intravenous rt-PA is relatively low. In our case, a high concentration of rt-PA may have influenced the successful dissemination of large thrombus in the whole internal cervical artery. Our case is of significance as it raises the question of unanswered efficacy of diverse injection protocol according to thrombus size and bleeding risk.

## 1 Introduction

Currently, recombinant tissue plasminogen activator (rt-PA) remains the only approved thrombolytic medication in acute ischemic stroke.^[1]^ The standard regimen of rt-PA is that total dose of 0.9 mg/kg of body weight, 10% of which was given as a bolus followed by constant infusion of the remaining 90% over 60 minutes. However, it is known that the rate of recanalization is low when the rt-PA is infused by this administration method, especially in occlusion of a large vessel such as an internal carotid artery (ICA).^[2]^ We report our experience of efficient recanalization of large arterial occlusion without hemorrhagic complication after rapid injection of rt-PA over 7 minutes.

## 2 Case report

**Figure 1:**
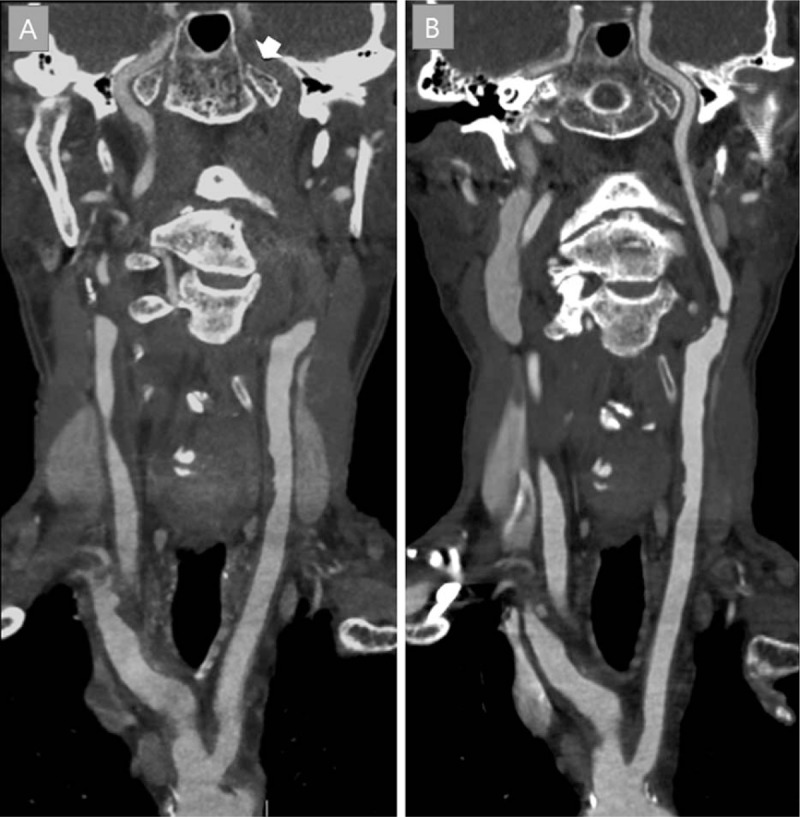
Computed tomography angiography before (A) and after (B) intravenous tissue plasminogen activator. Long segmental occlusion of the internal carotid artery (arrow) is recanalized after intravenous thrombolysis.

A 78-year-old man was admitted to our emergency department with right-sided hemiplegia and global aphasia that occurred an hour ago. His National Institutes of Health Stroke Scale (NIHSS) score of 21. Computed tomography (CT) of the brain performed 20 minutes after arrival revealed no hemorrhage or hypodense lesion. Ten percent of a total rt-PA dose was injected over 1 minute promptly after CT check. The remainder of rt-PA was designed to be infused for 60 minutes. Unexpectedly, during the study of CT angiography, administration of rt-PA was completed within 5 minutes with a significant disparity of the standard rt-PA protocol. Initial CT angiography showed nonvisualization of the ICA from carotid bifurcation to the middle cerebral artery (Fig. 1A). Because of the technical difficulties and bleeding risk, we concluded not to proceed with endovascular thrombectomy considering the long segmental thrombus of ICA. Two hours later, the patient began to regain strength in his right arm and leg (NIHSS 8). By the next day, he had only mild dysarthria and aphasia (NIHSS 2). Follow-up CT angiography revealed recanalized ICA and plaques on left proximal ICA with severe residual stenosis (Fig. 1B). It is thought that long segmental occlusion occurred from the proximal carotid artery to the middle cerebral artery due to the rupture of the carotid plaque. Magnetic resonance imaging scan showed multiple small diffusion-restricted lesions in the left basal ganglia and cerebral cortex. After 2 weeks, the patient underwent carotid endarterectomy for residual stenosis and was discharged without any neurological sequelae.

Informed consent was obtained from the patient for publication and accompanying images.

## 3 Discussion

In this case, the rt-PA was administered in a different protocol than the conventional method, resulting in the effective dissolution of the large thrombus without hemorrhagic complication. The conventional infusion protocol of rt-PA administration is established based upon NINDS trial in 1995 and has not changed in both clinical and research field.^[1,3,4]^ However, the dosing regimen used in this study was identical to the previous pilot study and did not consider the various administration methods. Although the study demonstrated the long-term clinical benefit of rt-PA compared to placebo, data related to early neurological improvement, which was the primary endpoint, did not reach statistical difference.^[1]^ Additional studies were conducted to extend the therapeutic time window or expand the eligible indication of rt-PA with identical protocol.^[3,5]^

There may be some limitations in applying the one identical thrombolytic protocol to all acute cerebral infarction. First, considering the short half-life of rt-PA, the primary purpose of intravenous thrombolysis is early recanalization. However, most previous studies have not shown that rt-PA administration is beneficial for early recanalization of large vessel occlusion compared to placebo. Successful revascularization was the most critical factor affecting clinical improvement after ischemic stroke in the previous study.^[6]^ Further effort to achieve early recanalization and an excellent clinical outcome is crucial in thrombolytic therapy.

Second, the rate of successful recanalization depends on the amount of thrombus. Long segmental thrombus located in a large vessel would be less likely dissolved compared to a small thrombus. In previous studies and clinical field, recanalization of large vessels has been observed up to 30% after only rt-PA administration.^[2]^ Because the ICA occlusion is more resistant to intravenous rt-PA than the MCA, only 4% of ICA occlusion is completely recanalized.^[7,8]^ The recanalization rate of whole ICA thrombus will be even lower than simple intracranial ICA. The presence of abundant blood clot in the whole ICA might prevent the delivery of an adequate dose of rt-PA to the thrombus. Nevertheless, the current protocol does not differentiate rt-PA administration between cerebral infarction due to small vessel disease and large artery occlusion. In our case, the high concentration of t-PA may have influenced the successful dissemination of large thrombus in the whole ICA.

In consideration of the administration of thrombolytic agents, another vital aspect is the risk of intracranial hemorrhage. In acute ischemic stroke, the size of cerebral infarction and the time from symptom onset are the main factors related to the risk of intracerebral hemorrhage.^[9,10]^ In a patient with large infarct core, low-dose rt-PA may be administered to reduce the risk of bleeding. The Enhanced Control of Hypertension and Thrombolysis Stroke Study was showed fewer symptomatic intracerebral hemorrhages with low-dose alteplase compared to full-dose.^[11]^ The longer the duration of thrombolytics administration from the onset of symptoms, the higher the risk of bleeding.^[12]^ In the ECASS III trial, intravenous rt-PA administration between 3 and 4.5 hours after the onset of symptoms significantly improved clinical outcome despite increased intracranial hemorrhage.

Conversely, patients with short time to onset of symptoms may have small infarct core and a lower risk of hemorrhage. In this case, increasing the dose or the administration rate as needed may not increase the bleeding risk to the effect. Despite these findings, there has been no study on the diversity of infusion protocol according to the risk of bleeding, and the guidelines only mention one method of administration.

Currently, endovascular thrombectomy is the most effective and essential treatment for a patient with large vessel occlusion. However, mechanical endovascular procedures require the facilities, equipment, and professional staff. Therefore, intravenous thrombolytic therapy is still an important treatment in many situations. Further studies are needed to increase the recanalization rate and early neurological recovery through intravenous rt-PA treatment. It is essential to make patient-tailored treatment decisions, such as recent studies of an endovascular thrombectomy or wake-up stroke that show effective results with different patient selections.^[5]^

This case is not an intended study, and there is a limit to generalization. However, our case is of significance as it raises the question of unanswered efficacy of various injection protocol according to thrombus size and bleeding risk and further discussion and well-designed studies are warranted.

## Author contributions

**Conceptualization:** Jaechun Hwang.

**Investigation:** Jaechun Hwang.

**Validation:** Jin-Sung Park.

**Writing – original draft:** Jin-Sung Park.

**Writing – review & editing:** Jaechun Hwang.
